# Understanding and tackling meat reduction in different cultural contexts: a segmentation study of Swiss and Vietnamese consumers

**DOI:** 10.3389/fpsyg.2024.1286579

**Published:** 2024-04-23

**Authors:** Mathilde Delley, Thanh Mai Ha, Franziska Götze, Evelyn Markoni, Minh Hai Ngo, Anh Duc Nguyen, Thi Lam Bui, Nhu Thinh Le, Bao Duong Pham, Thomas A. Brunner

**Affiliations:** ^1^Food Science & Management, School of Agricultural, Forest and Food Sciences (BFH-HAFL), Bern University of Applied Sciences, Zollikofen, Switzerland; ^2^Faculty of Economics and Rural Development, Vietnam National University of Agriculture, Hanoi, Vietnam; ^3^Department of Economics, The Swedish University of Agricultural Sciences, Uppsala, Sweden; ^4^Faculty of Accounting and Business Management, Vietnam National University of Agriculture, Hanoi, Vietnam; ^5^Department of Economics and Marketing, Fruit and Vegetable Research Institute, Hanoi, Vietnam; ^6^Bac Giang Agriculture and Forestry University, Bac Giang, Vietnam

**Keywords:** segmentation, consumer behaviour, Switzerland, Vietnam, meat consumption, reduction, emerging economies

## Abstract

**Objective:**

This study aims to disclose and compare meat consumer segments in Switzerland and Vietnam, which differ in terms of their socioeconomic and cultural settings (the former is a developed country, and the latter is an emerging one) to develop a set of segment-specific recommendations that might be applied to consumption in comparable contexts, that is, in other developed countries and other emerging economies.

**Methods:**

Data were collected through two online surveys: one for Swiss residents from randomly selected households and one for Vietnamese urban residents recruited via snowball sampling. The final sample size was *N* = 643 for Switzerland and *N* = 616 for Vietnam. Hierarchical cluster analyses followed by K-means cluster analyses revealed five distinct clusters in both countries.

**Results:**

Three clusters were common to both countries: meat lovers (21% in Switzerland and 19% in Vietnam), proactive consumers (22% in Switzerland and 14% in Vietnam) and suggestible consumers (19% in Switzerland and 25% in Vietnam). Two were specific to each country, namely traditional (19%) and basic (21%) consumers in Switzerland and confident (16%) and anxious (26%) consumers in Vietnam.

**Conclusion:**

Relying on voluntary actions, nudging techniques, private initiatives and consumers’ sense of responsibility will certainly be useful but will nevertheless be insufficient to achieve a planetary health diet within the given timeframe (the 2030 Agenda for Sustainable Development). Governments will have no choice but to activate all levers within their sphere of influence – including regulatory measures – and oblige private sector actors to commit to the measures imposed on them. A binding international agenda with common objectives and measures is a judicious approach. Unlike most previous studies, which focused on meat consumption intensity and frequency or diet type to segment consumers, our approach, based on psychographic profiles, allows the identification of segments that share common drivers and barriers and thus the development of better-targeted measures to reduce meat consumption.

## 1 Introduction

Historical and archaeological research has enabled us to better understand and follow the evolution of the place and role of meat in the human diet, from rare and special food reserved for feasting until the beginning of the twentieth century (Chiles and Fitzgerald, 2018), then a sign of wealth, prosperity and power, to everyday food in industrialised countries and emerging economies (Contreras, 2008). The limited availability and accessibility of meat (Marciniak, 2020), p. 243, as well as its symbolic value (Ulijaszek, 1991; Chiles and Fitzgerald, 2018; Marciniak, 2020), p. 208–210, aroused the interest and appetite of our ancestors and influenced our relationship with this special food.

### 1.1 Environmental load of meat consumption

However, the consumption and production of this special food cause considerable environmental burdens (Clune et al., 2017), and the current global population and its diet are putting enormous pressure on the planet’s resources, whose renewal is under threat (Willett et al., 2019). Humanity is already living on credit at the cost of future generations (Earth Overshoot Day, 2023). Considering the major consumption categories of EU households, food appears to be the main driver of the overall environmental impact (more than 30%) and in most of the impact categories, such as biodiversity, acidification, terrestrial, freshwater and marine eutrophication, land use, and water use (Sala et al., 2019). Taking a closer look at the root causes of the problem, meat production—especially red meat—appears to be a major contributor to the global warming potential attributable to people’s diets (Clune et al., 2017; Notarnicola et al., 2017; Neu, 2022). Beef generates the highest emissions and is responsible for a large proportion of the total impact of the diet, although it is consumed in smaller quantities than other types of meat (Notarnicola et al., 2017; Ferronato et al., 2021). Livestock production alone represented 14.5% of all human-induced greenhouse gas (GHG) emissions in 2005 (Gerber et al., 2013) and uses at least one-third of all arable land (Garnett, 2013; Mottet et al., 2017); thus, even if animal products represent a small proportion of one’s diet and contribute to food security, they have a substantial environmental impact. For example, the average daily consumption of an average omnivorous European diet of 150 g of meat and fish generates 37% of the carbon footprint, 38% of the water footprint and 44% of the ecological footprint (land and sea use) of this diet (Rosi et al., 2017). A study conducted in various developed and emerging Asian economies (Adhikari and Prapaspongsa, 2019) showed that reducing meat and animal product consumption would reduce the life-cycle impact of all diets under study—except for the Indian diet, which is traditionally low in meat—whilst maintaining a balanced diet. These figures highlight that meat production and consumption are a problem but also an important lever to tackle the environmental crisis. As a result, a substantial shift towards a plant-dominated universal diet has been proposed as a possible solution to preserve the health of both the planet and humans (Willett et al., 2019).

### 1.2 Health and meat consumption

Moderate meat consumption is associated with several positive health benefits. About 10% of the total energy intake (or 0.8 g/kg bodyweight) is considered an ideal protein intake for people aged older than 2 years. Protein quality is defined by the presence and relative proportion of essential amino acids. Animal protein sources show better amino acid profiles than most plant sources. Meat is also an important source of several micronutrients essential for health and disease prevention, such as iron, vitamin B12, selenium, zinc, niacin and phosphorus, some of which are found almost exclusively in animal products and show increased bioavailability within this matrix. Additionally, meat is an important source of bioactive nutrients, antioxidants and conjugated linoleic acids (Mann, 2018). A meta-analysis of Asian cohort studies showed a protective effect of poultry, red meat, and fish against all-cause mortality (Lee et al., 2013). Similarly, studies conducted in developing countries have shown a link between high meat intake and overall health improvements (Neumann et al., 2007; Mozumdar et al., 2009; Girard et al., 2012).

In contrast, the negative impacts of meat consumption on health have been widely discussed. Rather than the protein itself, other constituents of meat and ingredients added to processed meat products are thought to be responsible for the adverse health effects associated with meat consumption. Of these, the most frequently cited causes include the high proportion of saturated fat found in meat, carcinogens formed when meat is cooked at high temperatures, and sodium and preservatives added to processed meat (Willett et al., 2019). Moreover, several meta-analyses (Chen et al., 2013; Feskens et al., 2013; Abete et al., 2014) and large-scale studies (Sinha et al., 2009; Pan et al., 2011, 2012; Etemadi et al., 2017) have shown a clear association between red meat consumption and the risk of developing cardiovascular diseases and cardiovascular disease mortality. Based on an evaluation by the International Agency for Research on Cancer (Bouvard et al., 2015), the World Health Organization (WHO) has classified the consumption of red meat as possibly carcinogenic and the consumption of processed red meat as carcinogenic (WHO, 2015). A prospective cohort analysis conducted in the US showed that replacing animals with plant-based protein sources resulted in lower overall mortality and that a high intake of plant-based protein had a protective effect against cardiovascular mortality (Song et al., 2016). However, poultry and fish consumption are not associated with similar health risks (Abete et al., 2014).

### 1.3 Evolution of meat consumption

**Figure 1:**
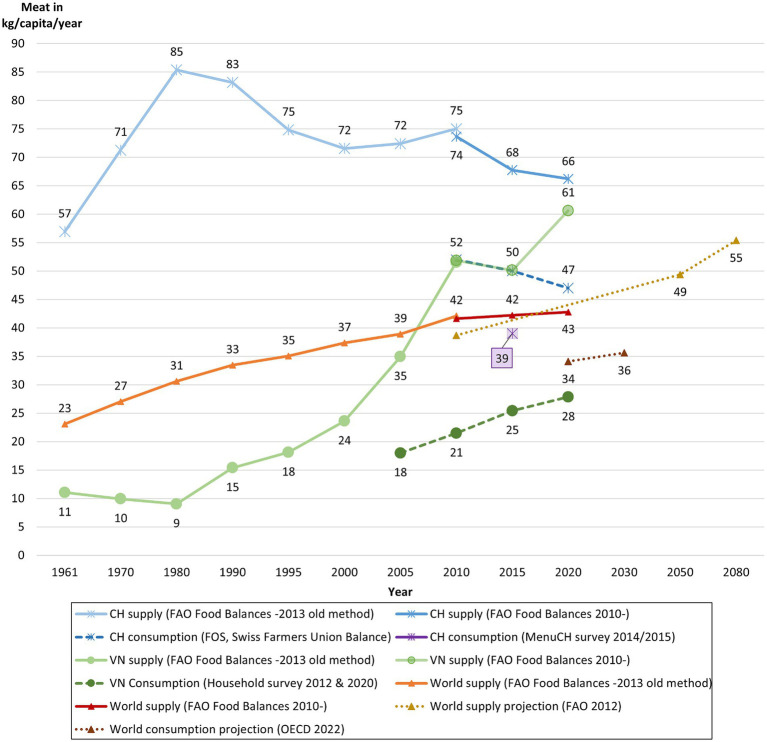
Meat supply and meat consumption in kg per year *per capita*. Sources: Alexandratos and Bruinsma (2012), GSO (2014), FSVO (2017), GSO (2022a), OECD (2022), FAO (2023a,b) and FSO (2023a).

Meat consumption and supply in Switzerland, an industrialised European country, and Vietnam, an emerging Asian country, present differing trends (Figure 1). The Swiss meat supply rose to 85 kg *per capita* per year until the 1980s and then declined to 66 kg by 2020. The first national nutritional consumer survey in 2014/2015 revealed a meat consumption *per capita* of 39 kg—one-third lower than the supply data. In contrast, Vietnamese supply remained at a modest level until the 1980s, after which it rose steadily to 61 kg *per capita* per year by 2020. Again, the consumer survey data indicate a much lower consumption (28 kg in 2020). Nonetheless, the descending trend in Switzerland versus the ascending trend in Vietnam remains robust and consistent across statistics. According to Whitton et al. (2021), increased meat consumption in Vietnam is associated with GDP growth. In Switzerland, concerns about health, the environment, animal welfare and improved availability of alternative proteins are the reasons for the decline in consumption (Whitton et al., 2021). The upward trend in Vietnam suggests that reducing individual meat consumption may be more challenging than in Switzerland, where meat reduction has already begun.

Segmentation studies can inform policy measures aimed at promoting meat reduction in countries with high meat consumption, such as Switzerland and Vietnam. The stimulation of consumer behaviour change towards meat reduction requires a comprehensive understanding of consumers’ current meat consumption behaviour, their willingness to change this behaviour, and their food choice motives. Segmentation analyses help identify distinct consumer segments that differ in terms of the factors described above. This information will allow the identification of policy interventions that account for consumer heterogeneity and are tailored for each consumer segment. Thus, the effectiveness of these interventions will be enhanced.

### 1.4 Evolution of the recommendations towards meat consumption

Nutritional recommendations have a fairly recent history and only a modest capacity to shape new eating habits. Nevertheless, they are interesting markers of the evolution of knowledge about nutrition, indicators of the priorities of the authorities that issue them, and indirect indicators of a population’s state of health and wealth. In 1935, the League of Nations published the first international standard dietary recommendations, followed by nutritional reference values (NRVs) in 1937 and the guiding principle of minimal food requirements in 1939 (Scholliers, 2013; Ridgway et al., 2019). Until at least the end of the 1970s, all existing national recommendations presented meat as a highly nutritious and healthy food and encouraged its consumption (Haughton et al., 1987; Santich, 2005; Ridgway et al., 2019). Depending on the national setting, the position of meat and animal proteins started to evolve and translate into adaptations of nutritional recommendations at some point between the late 1970s and the present day (Haughton et al., 1987; Truswell, 1987; Santich, 2005). In 1995, the Food and Agriculture Organization (FAO) and WHO conducted consultations to establish a scientific basis for the development of diet-based dietary guidelines (DBDGs). This has led to developing countries initiating the development of their own DBDGs. In the 1990s and the 2000s, NRVs were complemented by higher levels of intake in response to the growing problem of overweight and obesity (Ridgway et al., 2019).

The idea of a sustainable diet that preserves both the environment and human health was first developed in the 1980s by von Koerber (1981), who introduced the concept of a “wholesome diet” that aimed to place equal importance on the health, ecological, economic, social, and cultural dimensions of nutrition. It comprises a plant-based diet with minimal processed foods (von Koerber et al., 2017). A few years following the development of this idea, Gussow and Clancy (1986) highlighted that consumers need to be informed on how to make food choices that “not only enhance their own health but also contribute to the protection of our natural resources” and suggested new dietary guidelines that corresponded to this objective. However, these few attempts did not receive much attention, and the concept of a sustainable diet was neglected until the FAO developed its own definition of sustainable diets in 2010 (Ridgway et al., 2019):

Sustainable diets are those diets with low environmental impacts which contribute to food and nutrition security and to healthy life for present and future generations. Sustainable diets are protective and respectful of biodiversity and ecosystems, culturally acceptable, accessible, economically fair and affordable; nutritionally adequate, safe and healthy; while optimizing natural and human resources (Burlingame and Dernini, 2012), p. 294.

The importance of integrating environmental sustainability into dietary guidelines has since grown in popularity and was highlighted in the 2019 report of the EAT-Lancet Commission on healthy diets from sustainable food systems. The diet elaborated by this commission stands out for its low meat content (43 g/day for an intake of 2,500 kcal/day) compared to the current consumption of most people in developed countries (Willett et al., 2019). Since then, several countries have attempted to reformulate dietary guidelines to promote sustainable food systems, with varying degrees of success. Whilst Brazil, Canada, Qatar, Sweden and Germany have succeeded in doing so or officially committed themselves to achieving this in the next few months, Australia and the United States have fallen short of their targets, possibly because of intensive industry lobbying and media campaigns (Ridgway et al., 2019).

### 1.5 Food choice motives and meat consumption

Food choice motives have been widely studied, and measurement instruments have been developed, validated and updated (for some examples, see Brunsø and Grunert, 1995; Steptoe et al., 1995; Lindeman and Väänänen, 2000; Bell and Marshall, 2003; Verplanken and Orbell, 2003; Onwezen et al., 2019; Verain et al., 2021). Amongst the numerous constructs proposed, some appear to be particularly relevant to understanding meat consumption behaviour, meat perception and ongoing changes in consumption and perception. Motives can be divided into drivers and barriers; however, recent studies have highlighted that the same arguments can be considered for both drivers and barriers depending on the consumer segment asked (see Strässner and Hartmann, 2023 for a review). Thus, it seems appropriate to move away from this dichotomy and instead address motives in order of relative importance from the consumer’s point of view.

Endorsing this approach, health appears to be the overarching motive for reducing meat consumption as well as maintaining at least some meat consumption (Latvala et al., 2012; de Boer et al., 2017; Mylan, 2018; Lacroix and Gifford, 2019; Malek et al., 2019; Sanchez-Sabate et al., 2019; Fehér et al., 2020; Kemper, 2020; Collier et al., 2021, 2022; Kemper and White, 2021; Malek and Umberger, 2021a,b; Verain and Dagevos, 2022; Valli et al., 2023). Meat is sometimes perceived as an essential food that provides strength and vigour (de Koning et al., 2015; Stoll-Kleemann and Schmidt, 2017). Staying healthy and fit has been repeatedly reported to be more relevant to consumers than environmental concerns, which, nonetheless, are major arguments.

Environmental issues are more important for low-meat eaters, vegetarians and young people; they reinforce those already committed to reducing meat consumption but rarely bring about a change in diet on their own (de Boer et al., 2017; Sanchez-Sabate et al., 2019; Fehér et al., 2020; Hielkema and Lund, 2021; Kemper and White, 2021; Malek and Umberger, 2021a; Trewern et al., 2022; Verain and Dagevos, 2022; Verain et al., 2022; Valli et al., 2023). de Boer and Aiking (2021) suggest that pro-environmental food choices are not necessarily motivated by environmental concern but rather result from a complex process involving social motivation, such as being other-oriented and socially responsible. In the Asian context, the sustainability argument finds favour with consumers, mainly because it is associated with food that is free from pesticides, chemicals, and antibiotics (de Koning et al., 2015). Nevertheless, some consumers still express doubts about the relevance and effectiveness of reducing meat consumption, at least on an individual scale, to mitigate climate change and environmental depletion (Mullee et al., 2017; Marinova and Bogueva, 2019; Malek et al., 2019; Sanchez-Sabate et al., 2019; Collier et al., 2021; Valli et al., 2023). In Asia, quality and freshness seem to be the most important criteria for choosing food (de Koning et al., 2015). Markoni et al. (2023) confirmed this in their recent study.

Investigating the top food choice motives of different consumer groups, several authors (Latvala et al., 2012; Verain and Dagevos, 2022; Verain et al., 2022) have highlighted that meat eaters differ from vegetarians in that they prioritise egoistic motives such as health, taste, price and food safety over altruistic motives such as animal welfare and environmental sustainability. Meat attachment (Graça et al., 2015), a multidimensional construct that includes hedonism or the pleasure of eating meat, negatively affects the propensity to adopt a more plant-based diet. Sensory appeals such as taste complexity indeed belong to the arguments frequently cited in favour of meat consumption (Latvala et al., 2012; de Boer et al., 2017; Collier et al., 2021; Kemper and White, 2021; Verain and Dagevos, 2022; Halkier and Lund, 2023; Valli et al., 2023), whilst the enjoyment of eating has been evoked both in relation to the consumption of meat-based and meat-free meals (Mylan, 2018; Sanchez-Sabate et al., 2019; Fehér et al., 2020; Valli et al., 2023). Affordability also belongs to the arguments mentioned by the advocates of all diet types (de Boer et al., 2017; Lacroix and Gifford, 2019; Sanchez-Sabate et al., 2019; Kemper and White, 2021; Malek and Umberger, 2021a; Verain and Dagevos, 2022; Valli et al., 2023). Similar to nearly all dietary behaviours, habits and routines also play an important role when it comes to the place of meat in our diets and meals and more often than not act as a barrier or brake to the adoption of a low-meat diet (de Boer et al., 2017; Sanchez-Sabate et al., 2019; Hielkema and Lund, 2021; Halkier and Lund, 2023; Valli et al., 2023). Reducing and replacing meat in a diet is often a gradual process in which several deeply rooted daily practices (food provisioning, cooking and presentation of the dish) must be reshaped (Halkier and Lund, 2023). Such changes require new (culinary) skills (Lacroix and Gifford, 2019) and presuppose that convenient and healthy alternatives are widely available, which is not always the case for consumers (Mylan, 2018; Lacroix and Gifford, 2019; Sanchez-Sabate et al., 2019; Fehér et al., 2020; Collier et al., 2021, 2022; Hielkema and Lund, 2021; Kemper and White, 2021; Valli et al., 2023).

Animal welfare and rearing conditions (including issues related to the use of hormones and antibiotics), as well as slaughtering practices (de Boer et al., 2017; Mylan, 2018; Kemper, 2020; Verain and Dagevos, 2022; Valli et al., 2023), are topics raising questions related to not only ethical (Mylan, 2018; Lacroix and Gifford, 2019; Rosenfeld et al., 2020) but also food safety issues (Verain and Dagevos, 2022; Valli et al., 2023) that concern all groups of consumers—meat abstainers and heavy reducers in particular (Malek and Umberger, 2021a,b; Verain and Dagevos, 2022; Verain et al., 2022). Families also play an important role in food choices linked to meat consumption. The specific preferences and needs of other household members can be seen as both drivers and barriers to reducing meat consumption (de Boer et al., 2017; Mylan, 2018; Lacroix and Gifford, 2019; Fehér et al., 2020; Collier et al., 2022; Markoni et al., 2023; Valli et al., 2023), and a change in family relationships or household settings is sometimes regarded as an opportunity to adopt new eating behaviours (Mylan, 2018). Similarly, working environment and colleagues also influence food choices (Mylan, 2018). Finally, the social context in which one lives and society as a whole influence individual choices by determining what is perceived as normal (Sanchez-Sabate et al., 2019; de Boer and Aiking, 2021; Verain and Dagevos, 2022; Verain et al., 2022; Halkier and Lund, 2023), appropriate (de Boer et al., 2017; Mylan, 2018; Collier et al., 2022) or conforming (Lacroix and Gifford, 2019). Cultural heritage also plays a role in determining what is appropriate for a special occasion e.g., family celebration, religious holiday, party, barbecue, welcoming guests (Marinova and Bogueva, 2019; Markoni et al., 2023), for how to behave as guests, and as to what extent customs and traditions can be reinvented and adapted (de Boer et al., 2017; Mylan, 2018; Collier et al., 2021, 2022; Kemper and White, 2021).

Familiarity with food directly correlates with the intensity of meat consumption, with the heaviest consumers attaching greater importance to it (Malek and Umberger, 2021a). The question of identity expression through food choices seems to be of particular importance to vegetarians (Sanchez-Sabate et al., 2019; Rosenfeld et al., 2020; Collier et al., 2021; de Boer and Aiking, 2021; Hielkema and Lund, 2021; Amato et al., 2022). Finally, the provision of variety in a diet (de Boer et al., 2017; Mylan, 2018; Fehér et al., 2020; Kemper and White, 2021) and moral considerations (Verain and Dagevos, 2022) are also frequently cited motives in relation to meat consumption, with the latter being more specific to meat abstainers.

### 1.6 Segmentation of meat consumers

Studies conducted in recent decades have segmented consumers according to their level of meat consumption and yielded different results with various numbers of clusters. Three archetypal consumer clusters emerged in the first set of studies: meat lovers, meat reducers and meat abstainers. Latvala et al. (2012) used latent class analysis to segment Finnish consumers according to their past and planned changes regarding meat and vegetable consumption and ended up with three major clusters, including “no change,” “past change” and “ongoing change.” The “past change” cluster corresponded to meat reducers who had already achieved their goal, whereas the “ongoing change” cluster was further subdivided into four minor clusters according to the type of dietary change underway; two of these minor clusters could also be described as meat reducers. Dagevos and Voordouw (2013) asked Dutch consumers to identify themselves as vegetarians, flexitarians or meat eaters, and further split them into three subgroups according to how frequently they consumed meat-based dinners. Refining their approach, the same authors then employed cluster analysis on meat eaters with clustering variables, including not only meat consumption but also a mix of psychological variables such as food choice motives and meat perception. This approach allowed Dagevos and Voordouw (2013) to identify five clusters. Three of the clusters were considered flexitarians because of their moderate meat consumption, whilst two of them were regarded as regular meat eaters who did not intend to reduce their consumption. The five segments differed in their importance for meat consumption. Following the same approach, a second segmentation was performed in 2019, which yielded similar results. Despite a large increase in the share of the sample population identifying themselves as meat reducers, reported meat consumption and national statistics remained fairly constant for over a decade. Overall, a slow change towards moderating meat consumption, the growing importance of personal norms in relation to meat reduction and appreciation of meat-free meals were observed (Verain et al., 2022). Given the growing recognition of the psychological dimensions of meat consumption, most current studies in this area have adopted a similar approach using food-choice motives and more psychographic variables to segment meat consumers or describe segments. For instance, de Gavelle et al. (2019) classified French consumers into four dietary types based on their attitudes and beliefs towards protein sources: vegetarians, flexitarians, pro-flexitarians (meat reducers) and omnivores. The authors highlighted a gradual increase in the overall importance assigned to environmental impact, health impact, and animal welfare amongst the four dietary types found in their study, progressing from the less concerned omnivores to the very mindful vegetarians. Lacroix and Gifford (2019) used psychological drivers and barriers of meat reduction to segment Canadian consumers in a latent class analysis and identified two groups of meat eaters who differed in their perception of the number and intensity of barriers to meat reduction (moderate- and strong-hindrance meat eaters) and a group of meat reducers, including vegetarians and flexitarians. The authors proposed a hierarchy of meat reduction inhibitors, some of which are common to all groups, including meat reducers (e.g., seeking social conformity), others affecting both groups of meat eaters (e.g., belief that meat is necessary to stay healthy, lack of cooking skills to prepare meat-free meals) and one being specific to the strongly hindered meat eaters group (e.g., belief that alternatives are less convenient, food neophobia). Australian meat consumers were segmented according to their willingness to adopt different meat-reduction strategies in the future using discrete factor analysis, forming four groups: committed meat eaters building the largest group, consumers willing to reduce, prospective vegetarians or vegans, and undecided consumers (Malek et al., 2019).

Given that meat reduction is an urgent issue and arguing that neither vegetarians nor meat lovers will be the agents of change, a majority of the recent segmentation research has focussed on meat reducers or flexitarians to characterise them more precisely, rather than focussing on the whole population as in early studies. For example, Malek and Umberger (2021b) used latent class analysis to segment Australian flexitarians according to their meat-consumption frequency and meat-reduction intention. They identified five segments: one heavy meat reducer, one moderate meat reducer and three light meat reducers. The three light meat-reducers were differentiated according to the type(s) of meat consumed, and all five segments showed relatively modest intentions towards meat reduction. In particular, meat reducers were distinct from other groups in their perception of the health benefits of a meat-free diet and the relative importance they assigned to egoistic food-choice motives (e.g., price; Malek and Umberger, 2021a). Götze and Brunner (2021) used a hierarchical cluster analysis to group Swiss consumers based on their tendency to consume meat and meat alternatives. They identified six distinct clusters, ranging from the uncompromising meat eater to the meat rejector. Arnaudova et al. (2022) studied the Swiss student population and found four clusters that differed in their level of readiness to change meat-eating behaviours. More than half of the students surveyed were either willing to reduce their meat consumption or had already done so. Halkier and Lund (2023) applied latent class analysis to Danish flexitarians and identified four clusters according to perceived barriers to and facilitators of meat reduction. Their results suggest that flexitarians’ perceptions evolve over the course of their flexitarian journey, moving from a dominance of barriers to a dominance of facilitators to a possible loss of relevance of external influences.

### 1.7 The present research

Most segmentation studies on meat consumers have used intensity and frequency of meat consumption or diet type as segmentation criteria. This approach, which commonly ends up depicting the three archetypal consumer clusters, namely, meat lovers, meat reducers, and meat abstainers, fails to capture underlying consumer segments that may show some differences in their consumption patterns but share common perceptions of the drivers of and barriers to meat reduction. Moreover, related studies across cultures are limited; thus, the question of whether emerging economies differ from developed countries in relation to consumer segments and food choice motives amongst meat eaters has not been answered. The objective of this study is to identify and profile meat consumer segments in Vietnam and Switzerland based on perceptions and behaviour with regard to meat consumption, considering the diversity of the two groups in terms of sociocultural context and psychographic profile. The results will allow us to (a) draw parallels between the two countries and examine the specific features of the two contexts and (b) obtain a more detailed and informative view of the identified consumer segments. The final aim is to develop a set of recommendations for meat reduction that considers the diversity of consumer priorities whilst being tailored to different cultures. To the best of our knowledge, this is the first segmentation study on meat consumers that considers both behavioural and cultural aspects.

## 2 Materials and methods

This study is part of a larger research project that investigates sustainable food consumption behaviours in Switzerland and Vietnam. To this end, we designed a survey and sent it to large samples of both populations. Whilst the questionnaire design and data analysis were jointly conducted, the recruitment methods and data collection tools were different for the two countries and are therefore described separately.

### 2.1 Data collection and sample

#### 2.1.1 Switzerland

The research team selected 33 ZIP code areas spread across the German- and French-speaking regions, covering all cantons (except Italian-speaking Ticino), and a mix of urban, semi-urban, and rural areas. This selection aimed to reflect the diversity of the Swiss population in term of language, region, and population density. After that, the sample was recruited by flyers being sent to all households situated in the areas with the selected ZIP codes. The flyer briefly described the study and its scientific purpose and provided a simple hyperlink to participate in the online survey. In each household, whichever person aged over 16 who would next celebrate their birthday was invited to take part in the survey (as a means to randomise participants across households). This procedure, which was developed with the aim of obtaining a sample as diverse as possible and potentially representative of the adult Swiss population, has been used successfully in several previous studies (Tüfer and Brunner, 2023; Brunner et al., 2023; Lucas and Brunner, 2024). The very high Internet penetration rate in Swiss households [89% of the population aged 14+ were regular users in Winter 2019/20, increasing by about 1% each year (FSO, 2022c)], convinced us that this method was in line with our objective of achieving a representative sample. Data were collected over 3 weeks between November and December 2022. The EFS survey tool offered by Unipark was used to programme the questionnaire and collect data. Of the 29,992 flyers sent out, 933 households completed the online questionnaire (3.1% response rate); all questions except for those on income were mandatory. Participants who dropped out before starting to answer the socio-demographic questions (placed at the end of the questionnaire) and those who failed the consistency check were removed, leaving 643 valid cases for analysis. The sample comprised 643 cases after cleaning the data. Participants who indicated that they were vegetarian or vegan were not asked to answer questions directly related to meat consumption and reduction. As these questions were used to build some of the segmentation variables, the sample used in the analyses included only omnivores and flexitarians and was smaller (*N* = 570).

#### 2.1.2 Vietnam

The target population of the sample was intentionally limited to urban consumers as meat reduction, which is the main topic of the questionnaire, is less relevant in rural areas, where food insecurity and children’s undernutrition are more prevalent (Vu and Rammohan, 2022). Amongst the five largest cities in Vietnam, we selected three for the survey: Hanoi, Haiphong and Ho Chi Minh City, which are amongst the most developed cities in Vietnam. The first and second were allocated to the north, and the third to the south of the country. A snowball approach was used to recruit participants since it appears to be the most suitable approach, given the available resources. This sampling method has been employed by some studies on consumer behaviour in sustainable consumption (Francis and Sarangi, 2022; Mishra and Kulshreshtha, 2023). The starting sample was approached using professional networks. The research team sent a link to the survey and an invitation letter to the managers of companies, consumer associations, and parent associations of schools in the three selected cities. The managers were then asked to forward the link and letter to their employees and members via social media platforms such as Zalo and Facebook; participants were invited to share the link within their own networks. An online survey was considered an appropriate means to reach the target population and disseminate the survey as about 79% of the population aged 18 or older are Internet users and about 71% of the total population uses at least one social media platform (Kemp 2023). These figures are expected to be even higher amongst urban populations. Only individuals aged 18+ years and then living in the urban districts of Ha Noi, Haiphong and Ho Chi Minh City were invited to participate in the survey. Data were collected over 4 weeks between November and December 2022. The questionnaire was programmed using the survey tool Netigate and formatted to be completed on smartphones, which are commonly used to access the Internet in urban regions. All the questions were mandatory. Participants who failed the consistency check and those who spent less than 13 min on the questionnaire (a timeframe judged as the minimum time needed to properly answer it) were excluded, leaving 616 valid cases for analysis after data cleaning.

### 2.2 Questionnaire

The questionnaire covered different topics related to sustainable eating practices and comprised five sections. Diet type (omnivorous, flexitarian, vegetarian and vegan) and meat consumption were queried in the first section. Meat consumption was assessed using two questions: *(1) How many times a week do you eat meat on average?* and *(2) When you eat meat, how much meat do you eat on average?* For the first question, a range from less than one to more than 20 was provided, whilst for the second question, a range from 25 g to 250 g (e.g., 25 g, 50 g, 75 g, … 200 g, 250 g) was given. Pictures representing several sizes of meat portions of several meat types (e.g., beef, pork, poultry, processed meat) were included to facilitate accurate evaluation. Respondents were reminded that the two questions asked about all types of meat included in- and out-of-house consumption, take-away, meat in ready meals and processed meats. Fish and seafood were neither represented nor mentioned and were thus implicitly not considered as meat. Average weekly meat consumption was then computed by multiplying the frequency of meat consumption by the average portion size.

In the following sections, participants were asked to indicate, on 6-point Likert scales, whether they agreed or were concerned about a series of statements relating to meat consumption, perception of meat, and food choice motives. These statements were related to the latent constructs used as segmentation variables and were measured using scales. Scales covering (1) meat safety concerns, (2) meat attachment, (3) perceived health risk of meat overconsumption, (4) self-efficacy in meat reduction, (5) hindering familial influence, (6) intention to reduce meat consumption, (7) animal welfare, (8) pro-environmental attitude, and (9) preference for local and seasonal food were included. Respondents indicated their level of concern (1 = *not at all concerned* to 6 = *extremely concerned*) to the first construct, their degree of agreement (1 = *strongly disagree* to 6 = *strongly agree*) with the next five constructs, and the level of importance (1 = *not important at all* to 6 = *very important*) they attached to the remaining three constructs. Meat attachment reflects a positive bond to meat, whilst perceived health risk captures perceived health risks associated with meat consumption. Self-efficacy refers to consumers’ perception of their ability to reduce meat intake. Hindering familial influence reflects the negative influence of other family members who have unhealthy eating habits. Table 1 provides detailed information on the items used for each construct, construct internal reliability, and the source. The questionnaire ended with questions on socio-demographics, some of which were included in our analysis as profiling variables. It is worth noting that some sections of the questionnaire included questions relating to other aspects of sustainable food consumption that were not used in the present study (food waste, increased vegetable consumption).

**Table 1:** Segmentation items per construct, including internal reliability coefficients and sources.

Scales and items	Source
1. Meat safety concerns (Cronbach’s *α* = 0.81)	Ha et al. (2019) ^a^
*Indicate how you are concerned about the following: …*	
Hormones (growth stimulators) and drugs (antibiotics) residues in meat	
Bacterial contamination of meat due to lack of freshness or improper handling	
Preservatives in processed meat	
2. Meat attachment (Cronbach’s *α* = 0.77)	
*Indicate how strongly you agree with the following statements in relation to your meat consumption…*	
I eat meat because it is part of my daily eating habits	Arnaudova et al. (2022) ^a^
Eating meat provides me irreplaceable sensory pleasure	Arnaudova et al. (2022) ^a^
Meat is the centrepiece of all important meals, e.g., at family or friends’ gatherings	New
3. Perceived health risk of overconsuming meat (Cronbach’s *α* = 0.70)	
*Indicate how strongly you agree with the following statements in relation to your meat consumption…*	
A high meat consumption is not good for my health because meat is high in saturated fat and cholesterol	Arnaudova et al. (2022) ^a^
A high meat consumption would make me overweight	New
A high consumption of processed meat is bad for my health	Arnaudova et al. (2022) ^a^
4. Self-efficacy of meat reduction (Cronbach’s *α* = 0.80)	New
*Indicate how strongly you agree with the following statements in relation to your meat consumption…*	
I can put together different menus that contain less meat and are still healthy	
I am able to find alternatives to reduce my meat consumption	
I have enough knowledge to replace some of the meat in the meals I eat/prepare	
5. Hindering familial influence (Cronbach’s *α* = 0.75)	Markoni et al. (2023) ^a^
*Indicate how strongly you agree with the following statements in relation to your meat consumption…*	
Other members of my household like to eat meat and I find it difficult to reduce my meat consumption under these circumstances	
Other members of my household do not like vegetables, and this prevents me from eating more vegetables	
The specific needs of other members of my household (e.g., children, sick people) prevent me from adopting new eating habits	
6. Intention to reduce meat consumption (Cronbach’s *α* = 0.97)	(Prochaska et al., 2015), p. 98^a^
*Indicate how strongly you agree with the following statements in relation to your meat consumption…*	
It is likely that I will eat less meat in the next few months	
I intend to reduce the frequency with which I eat meat over the next few months	
I plan to reduce the amount of meat I eat over the next few months	
7. Animal welfare (Cronbach’s *α* = 0.92)	Lindeman and Väänänen (2000) and Verain et al. (2021)
*How important is it to you that the food you eat on a typical day…?*	
… Is produced without animals being in pain	
… Is produced with respect for animal rights	
… Is produced in an animal-friendly way	
8. Pro-environmental attitude (Cronbach’s *α* = 0.93)	Lindeman and Väänänen (2000) and Verain et al. (2021)
*How important is it to you that the food you eat on a typical day…?*	
… Is produced in a way without disturbing the balance of nature	
… Is prepared in an environmentally friendly way	
… Is produced in an environmentally friendly way^b^	
9. Preference for local and seasonal food (Cronbach’s *α* = 0.82)	Verain et al. (2021)
*How important is it to you that the food you eat on a typical day…?*	
… Is a local/regional product	
… Is a seasonal product	
… Comes from close by (little transport distance)	

a Items inspired from the mentioned source.

b Item slightly rephrased.

### 2.3 Data analysis

The datasets from Switzerland and Vietnam were aligned prior to merging, and reliability analysis was performed on the nine scales used for segmentation. These included meat safety concerns, meat attachment, perceived health risk of meat overconsumption, self-efficacy in meat reduction, hindrance of familial influence, intention to reduce meat consumption, animal welfare attitudes, pro-environmental attitudes and preferences for local and seasonal food. The results were satisfactory to very good (Cronbach’s *α* = 0.70–0.97). The mean scores of the three corresponding items were computed for each scale and saved for further analysis. A summary of the nine scales, corresponding items and reliability coefficients (Cronbach’s *α*) is presented in Table 1. We tested the nine constructs for multicollinearity (Pearson correlation coefficient >0.8), which was considered satisfactory. As the aim of the study was to compare the behaviours of the two countries, the datasets from Switzerland and Vietnam were considered separately and analysed in parallel once the scales were built. In the first stage, the nine constructs were used to conduct hierarchical cluster analyses using the nearest neighbour (also called single linkage) method and the squared Euclidean distance as a distance measure. According to Backhaus et al. (2021), p. 534 and Sarstedt and Mooi (2019), p. 334, this procedure is recommended to disclose potential outliers before conducting hierarchical cluster analysis using Ward’s method. Following this approach, we identified one outlier in the Swiss dataset and two outliers in the Vietnamese dataset; these were flagged and excluded from further analyses. Then, hierarchical cluster analyses using Ward’s method and the squared Euclidean distance were conducted on standardised data using the same nine constructs as the segmentation variables. We assessed the solutions between two and eight clusters using three criteria to determine the ideal number of clusters: visual inspection of the dendrogram, percentage change in the clustering coefficients of the agglomeration schedule and the variance ratio criterion (VRC). Neglecting the two-cluster solution (Backhaus et al., 2021), the evaluation procedure led to the selection of the five-cluster solution for the Swiss dataset and both the four-and five-cluster solutions for the Vietnamese dataset for further analysis. Next, we assessed the stability of the clustering solutions by performing a K-means clustering analysis using previously determined cluster numbers and centres, following the procedure described by Sarstedt and Mooi (2019), section 9.4.1.4. We then compared cluster affiliations between the results obtained using Ward’s and K-means methods. In the Swiss sample, 29% of cases changed the cluster affiliation between the two methods. As this figure exceeded 20%, we considered and analysed other solutions, which, however, did not yield better results; thus, the five-cluster solution was maintained. In the Vietnamese sample, both the four- and five-cluster solutions yielded very similar results with 77–78% affiliation overlaps and were thus retained for further analyses. These solutions were subjected to statistical analyses using general linear models (GLMs) and contrast analyses. Most of the segmentation variables showed unequal variance; thus, we used both analysis of variance and robust tests (Welch and Brown-Forsythe) to evaluate the distinctiveness of clusters across the different solutions. In the Swiss sample, the five clusters differed significantly (*p* < 0.001) across the nine segmentation variables. Furthermore, contrast analyses revealed that each cluster differed significantly from the other four clusters in at least seven of the nine segmentation variables. For the Vietnamese sample, both the four- and five-cluster solutions differed significantly (*p* < 0.001) across the nine segmentation variables. Contrast analyses revealed slightly more distinct clusters in the five-cluster solution, confirming the presence of five clusters and justifying its selection. For both samples, GLMs were used to analyse and describe the five-cluster solution regarding additional food-choice motives, information about diet and meat consumption, and socio-demographic features.

## 3 Results

### 3.1 Sample characteristics

The Swiss sample was older and lived in larger households than the Swiss population overall. People of Swiss nationality and those with a high level of education were overrepresented, and these two features could be correlated. The characteristics of the sample population are summarised in Table 2. The Vietnamese sample was highly educated and had a higher proportion of full-time workers than the overall Vietnamese population, both of which are likely related to the selected recruitment channels. Details of the sample and comparison with the Vietnamese population are presented in Table 3.

**Table 2:** Socio-demographic characteristics of the sample compared with the resident population of Switzerland.

Characteristics	Sample population	Swiss population
**Gender**		
Men	44%	50%
Women	55%	50%
**Age groups**		
16–19	3%	5%
20–39	21%	31%
40–59	38%	34%
60–79	34%	24%
≥80	4%	6%
**Nationality**		
Swiss	91%	74%
Other	9%	26%
**Residence area** ^*^		
Urban	27%	63%
Semi-urban	35%	22%
Rural	38%	15%
**Education** ^**^		
None/compulsory	2%	18%
sec. Professional	19%	33%
sec. General	9%	9%
tert. Professional	27%	17%
tert. University	43%	23%
**Household size**		
1 person	19%	37%
2 persons	45%	33%
3 persons	13%	13%
4 persons	16%	12%
5 persons	5%	5%
≥6 persons	2%	1%
**Gainful employment** ^***^		
Full time (≥90%)	34%	38%
Part time (50–89%)	21%	13%
Part time (<50%)	9%	9%
None	36%	39%

^*^Federal Statistical Office (FSO) territorial typology may differ from the perception of the respondents; ^**^Resident population ≥25 years; ^***^Resident population ≥15 years. Sources: FSO (2021, 2022a,b, 2023a,b,c,d).

**Table 3:** Socio-demographic characteristics of the sample compared with the resident population of Vietnam.

Characteristics	Sample population	Vietnamese population
**Gender**		
Men	41%	50%
Women	59%	50%
**Age groups**		
15–19	2%	9%
20–39	52%	41%
40–59	40%	33%
60–79	6%	14%
≥80	0%	3%
**Nationality** ^*^ **(urban region)**		
Vietnamese	100%	98%
Other	0%	2%
Living area^**^		
Urban	100%	37%
Rural	0%	63%
**Urban area**		
Hanoi	34%	n/a
Hai Phong	32%	n/a
TP Ho Chi Minh	34%	n/a
**Highest education (urban region)**		
No technical/professional education	4%	64%
of which none	0%	7%
of which primary	0%	15%
of which secondary	4%	41%
sec. Professional (vocational ed.)	3%	7%
sec. General	6%	5%
tert. Professional	9%	6%
tert. University	77%	19%
**Household size (urban region)**		
1 person	3%	13%
2–4 persons	63%	66%
≥5 persons	35%	21%
**Gainful employment**		
Full time	70%	47%
Part time	19%	22%
None	11%	31%

^*^Resident population ≥15 years; ^**^the questionnaire was explicitly aimed at urban residents; thus, the sample population is expected to be 100% urban. Sources: GSO (2021), International Labour Organization (2021) and GSO (2022a,b,c).

### 3.2 Description of the segments

Cluster analysis revealed five clusters in both the Swiss and Vietnamese samples. The three clusters common to both countries include meat lovers (20% in Switzerland and 19% in Vietnam), proactive (22% in Switzerland and 14% in Vietnam) and suggestible (19% in Switzerland and 25% in Vietnam). The remaining two were country-specific: traditional (19%) and basic (20%) were identified for the Swiss sample, whilst confident (16%) and anxious (26%) were identified for the Vietnamese sample. The mean scores for the segmentation variables and the contrast analyses results are summarised in Tables 4, 5. In Tables 6–9, the segments are further characterised according to their diet, meat consumption, additional food choice motives and socio-demographic features.

**Table 4:** Mean scores over the segmentation variables and contrast analysis results by clusters within the Swiss sample.

	Meat lovers (20%) *n* = 117	Proactive (22%) *n* = 124	Suggestible (19%) *n* = 106	Traditional (19%) *n* = 106	Basic (20%) *n* = 117	Total (100%) *N* = 570
Meat safety concerns^1 ***^	3.40^D^	4.10	4.82^D^	**4.85** ^**D**^	*2.55^D^*	3.91
Meat attachment^2 ***^	**3.97** ^**D**^	*2.27^D^*	3.84^D^	3.67	3.92^D^	3.51
Perceived health risk of overconsuming meat^2 ***^	3.49^D^	4.05^D^	**4.39** ^**D**^	3.75	*2.81^D^*	3.69
Self-efficacy of meat reduction^2 ***^	*4.39^D^*	**5.60** ^**D**^	5.02	5.07^D^	4.58^D^	4.94
Hindering familial influence^2 ***^	2.14	1.72^D^	**2.77** ^**D**^	*1.70^D^*	1.86^D^	2.03
Intention to reduce meat consumption^2 ***^	2.65^D^	4.24^D^	**4.40** ^**D**^	*2.03^D^*	2.32^D^	3.14
Animal welfare^3 ***^	*3.64^D^*	**5.55** ^**D**^	5.23^D^	5.23^D^	5.20^D^	4.96
Pro-environmental attitude^3 ***^	*3.84^D^*	**5.61** ^**D**^	5.33^D^	5.37^D^	5.06	5.04
Preference for local and seasonal food^3 ***^	*4.12^D^*	**5.36** ^**D**^	4.85	5.16^D^	4.96	4.89

^***^p < 0.001; bold = the highest score for the variable; italics = the lowest score for the variable; ^D^Indicates the distinctiveness of the cluster against all others for the selected scale. ^1^Mean scores on a 6-point Likert scale, 1 = “not concerned at all” to 6 = “extremely concerned”; ^2^Mean scores on a 6-point Likert scale, 1 = “strongly disagree” to 6 = “totally agree”, ^3^Mean scores on a 6-point Likert scale, 1 = “not important at all” to 6 = “very important.”.

**Table 5:** Mean scores over the segmentation variables and contrast analysis results by clusters within the Vietnamese sample.

	Meat lovers (19%) *n* = 113	Proactive (14%) *n* = 84	Suggestible (25%) *n* = 145	Confident (16%) *n* = 97	Anxious (26%) *n* = 151	Total (100%) *N* = 590
Meat safety concerns^1 ***^	*4.47^D^*	5.00^D^	4.96^D^	4.56^D^	**5.13** ^**D**^	4.85
Meat attachment^2 ***^	4.61^D^	*2.48^D^*	**4.63** ^**D**^	4.30^D^	4.57^D^	4.25
Perceived health risk of overconsuming meat^2 ***^	4.22	4.27	**4.77** ^**D**^	*3.52^D^*	4.75^D^	4.38
Self-efficacy of meat reduction^2 ***^	4.42^D^	4.77	4.89^D^	*4.08^D^*	**4.95** ^**D**^	4.67
Hindering familial influence^2 ***^	3.21	2.71^D^	**4.40** ^D^	2.86*^D^*	*2.57^D^*	3.21
Intention to reduce meat consumption^2 ***^	3.81^D^	**4.82** ^**D**^	4.75^D^	*2.42^D^*	4.56^D^	4.15
Animal welfare^3 ***^	*2.86^D^*	4.71^D^	4.92^D^	4.33	**5.03** ^**D**^	4.43
Pro-environmental attitude^3 ***^	*4.01^D^*	5.19^D^	5.21^**D**^	5.04	**5.43** ^D^	5.01
Preference for local and seasonal food^3 ***^	*3.50^D^*	4.62^D^	**4.84** ^**D**^	4.34	4.47	4.37

^***^p < 0.001; bold = the highest score for the variable; italics = the lowest score for the variable; ^D^Indicates the distinctiveness of the cluster against all others for the selected scale. ^1^Mean scores on a 6-point Likert scale, 1 = “not concerned at all” to 6 = “extremely concerned”; ^2^Mean scores on a 6-point Likert scale, 1 = “strongly disagree” to 6 = “totally agree”, ^3^Mean scores on a 6-point Likert scale, 1 = “not important at all” to 6 = “very important.”.

**Table 6:** Diet type, meat consumption and food choice motives by cluster within the Swiss sample.

	Meat lovers (20%) *n* = 117	Proactive (22%) *n* = 124	Suggestible (19%) *n* = 106	Traditional (19%) *n* = 106	Basic (20%) *n* = 117	Total (100%) *N* = 570
**Self-declared diet**						
Omnivorous^***^	**79%**	*31%*	71%	71%	**79%**	66%
Flexitarians (mainly vegetarian with occasional meat or fish consumption)^***^	*21%*	**69%**	29%	29%	*21%*	34%
Average weekly meat consumption (kg)^***^	**0.71**	*0.22*	0.49	0.51	0.63	0.55
**How important is it to you that the food you eat on a typical day…?** ^1^						
… is nutritious^***^	*4.16*	4.86	4.80	**5.00**	4.62	4.69
… is affordable^***^	3.70	*3.31*	**3.85**	3.50	3.33	3.54
… is good for my health^***^	*4.42*	**5.27**	5.20	5.25	4.74	4.98
… has a very high level of food safety^***^	*4.00*	4.76	4.87	**4.94**	4.39	4.59
… is in line with my culture^*^	*3.12*	3.37	3.40	**3.86**	3.26	3.40

^*^p < 0.05 and ^***^p < 0.001; bold = the highest score for the variable; italics = the lowest score for the variable; ^1^Mean scores on a 6-point Likert scale, 1 = “not important at all” to 6 = “very important.”.

**Table 7:** Diet type, meat consumption and food choice motives by cluster within the Vietnamese sample.

	Meat lovers (19%) *n* = 113	Proactive (14%) *n* = 84	Suggestible (25%) *n* = 145	Confident (16%) *n* = 97	Anxious (26%) *n* = 151	Total (100%) *N* = 590
**Self-declared diet**						
Omnivorous^***^	**100%**	*88%*	98%	98%	97%	96%
Flexitarians (mainly vegetarian with occasional meat or fish consumption)^***^	*0%*	**12%**	2%	2%	3%	4%
Average weekly meat consumption (kg)^***^	**1.04**	*0.40*	0.72	0.77	0.79	0.74
**How important is it to you that the food you eat on a typical day…?** ^1^						
… is nutritious^***^	*4.85*	5.39	5.32	5.15	**5.45**	5.23
… is affordable^***^	*4.71*	4.99	**5.12**	4.87	5.09	4.96
… is good for my health^***^	*5.07*	5.58	5.46	5.27	**5.63**	5.40
… has a very high level of food safety^***^	*5.03*	5.42	5.42	5.26	**5.62**	5.35
… is in line with my culture^***^	*3.80*	4.60	**4.92**	4.40	4.23	4.39

^***^p < 0.001; bold = the highest score for the variable; italics = the lowest score for the variable; ^1^Mean scores on a six-point Likert scale, 1 = “not important at all” to 6 = “very important.”.

**Table 8:** Socio-demographic features by cluster within the Swiss sample.

	Meat lovers (20%) *n* = 117	Proactive (22%) *n* = 124	Suggestible (19%) *n* = 106	Traditional (19%) *n* = 106	Basic (20%) *n* = 117	Total (100%) *N* = 570
**Gender** ^***^						
Female	*41%*	**69%**	59%	57%	50%	55%
Male	**59%**	*31%*	41%	43%	50%	45%
**Nationality** ^*^						
Swiss (or binational)	**95%**	91%	*86%*	**95%**	92%	91%
Non-Swiss	*5%*	9%	**14%**	*5%*	8%	9%
**Living area** ^*^						
Rural	35%	*31%*	37%	**47%**	42%	38%
Semi-urban	38%	31%	**46%**	*28%*	32%	35%
Urban	27%	**38%**	*16%*	25%	26%	27%
**Education** ^*^						
Secondary	**3%**	*1%*	2%	2%	**3%**	2%
Vocational education	22%	*13%*	17%	22%	**23%**	19%
High school	**11%**	10%	9%	*6%*	8%	9%
Higher technical/professional education	23%	27%	*18%*	**36%**	29%	27%
University/university of applied science	41%	49%	**54%**	*34%*	37%	43%
**University degree** ^*^						
No university degree	59%	51%	*46%*	**66%**	63%	57%
University degree	41%	49%	**54%**	*34%*	37%	43%

^*^p < 0.05 and ^***^p < 0.001; bold = the highest score for the variable; italics = the lowest score for the variable.

**Table 9:** Socio-demographic features by cluster within the Vietnamese sample.

	Meat lovers (19%) *n* = 113	Proactive (14%) *n* = 84	Suggestible (25%) *n* = 145	Confident (16%) *n* = 97	Anxious (26%) *n* = 151	Total (100%) *N* = 590
**University degree** ^*^						
No university degree	18%	21%	23%	*13%*	**29%**	22%
University degree	82%	79%	77%	**87%**	*71%*	78%
**Household income** ^**^						
<5 m VND	2%	**5%**	4%	*0%*	1%	2%
5–10 m VND	*3%*	**12%**	8%	4%	7%	7%
10–18 m VND	*17%*	26%	23%	**33%**	22%	24%
18–32 m VND	**41%**	39%	41%	*30%*	34%	37%
32–52 m VND	24%	*8%*	14%	**25%**	24%	19%
52–80 m VND	8%	5%	8%	*3%*	**11%**	7%
>80 m VND	**6%**	5%	*1%*	5%	*1%*	3%

^*^p < 0.05 and ^**^p < 0.01; bold = the highest score for the variable; italics = the lowest score for the variable.

#### 3.2.1 The meat lovers

##### 3.2.1.1 Switzerland (20%)

As shown in Table 4, Swiss meat lovers are strongly attached to meat and place less importance on environmental issues, animal welfare and local and seasonal foods than other consumers. Health and safety are also of minimal concern. Furthermore, they do not feel confident in their ability to adopt a low-meat diet. Unsurprisingly, Swiss meat lovers show the highest meat consumption (0.71 kg/week) amongst the Swiss sample (Table 6); this segment is clearly male-dominated (59%; Table 8).

##### 3.2.1.2 Vietnam (19%)

Vietnamese meat lovers share many characteristics with their Swiss counterparts, although they have a more balanced gender distribution. They distinguish themselves from other segments by the low value they assign to animal welfare, the environmental impacts of food production (Table 5), and their particularly high meat consumption (1.04 kg/week; Table 7). Unlike most of their compatriots, they place little emphasis on culturally appropriate food choices (Table 5). Vietnamese meat lovers also distinguish themselves based on higher household incomes (Table 9).

#### 3.2.2 The proactive

##### 3.2.2.1 Switzerland (22%)

On the opposite side of the consumer profile spectrum, consumers are proactive. Proactive consumers living in Switzerland (hereafter called “Swiss consumers” for ease of readability) are not attached to meat consumption; they do attach, however, great importance to the impact of their food choices on the environment and on their health (Tables 4, 6). They pay attention to the locality and seasonality of food production and are concerned about animal welfare (Table 4). They had the lowest meat consumption (0.22 kg/week) across all segments (Table 6) and could effortlessly replace or renounce meat (Table 4). Most participants (69%) described themselves as flexitarians (Table 6). Food prices were not of major importance, but the nutritional aspects of the food were highly valued. This segment is clearly female-dominated (69%), highly educated, urban and comprises a higher share of people holding Swiss nationality (Table 8).

##### 3.2.2.2 Vietnam (14%)

The Vietnamese proactive are similar to their Swiss counterparts but have a more moderate profile. They distinguish themselves from their fellow citizens by their lower attachment to meat (Table 5), the presence of a higher percentage of flexitarians, their moderate meat consumption (0.44 kg/week) (Table 7) and their highest intention to further reduce said consumption (Table 5). They have the highest percentage of respondents in the two lowest income levels (Table 9). Like their Swiss counterparts, they place importance on nutritious and healthy foods (Table 7).

#### 3.2.3 The suggestible

##### 3.2.3.1 Switzerland (19%)

Suggestible Swiss consumers clearly intend to reduce their meat consumption but are also sensitive to the food choices of those around them, which negatively influences their ability to adopt new eating habits. They are concerned about the health consequences of excessive meat consumption (Table 4). Suggestible Swiss consumers form the most price-sensitive segment amongst Swiss consumers, show the highest share of people holding a university degree and comprise the highest share of foreigners (Tables 6, 8).

##### 3.2.3.2 Vietnam (25%)

Vietnamese suggestible consumers share several characteristics with their Swiss counterparts. For instance, they have a high intention to reduce meat consumption but are influenced by their relatives (Table 5). Their food choices are influenced not only by the choices and preferences of their families but also by those of society in general (Table 7). Furthermore, similar to their Swiss counterparts, they place importance on animal welfare and the environment. Vietnamese suggestible consumers are also price sensitive.

#### 3.2.4 The traditional

##### 3.2.4.1 Switzerland (19%)

Traditional consumers are distinguished by their low intention to reduce meat consumption (Table 4). They are concerned about food safety in general and meat safety in particular. They are also the most concerned about eating nutritious and culturally appropriate foods amongst the Swiss (Tables 4, 6). Traditional consumers are dominated by Swiss nationals and people living in the countryside. This segment has the lowest proportion of people with university degrees.

#### 3.2.5 The basic

##### 3.2.5.1 Switzerland (20%)

Basic consumers are convinced meat eaters; they have few concerns about meat safety and do not see any issues related to meat overconsumption (Table 4). Basic consumers show the lowest level of education amongst Swiss consumers (Table 8).

#### 3.2.6 The confident

##### 3.2.6.1 Vietnam (16%)

Confident consumers have the lowest concern about the health risks linked to meat overconsumption and feel barely affected by meat and food safety (Tables 5, 7); this confidence, coupled with their low-self efficacy in meat reduction, may translate into their low intention to reduce their meat consumption (Table 5). This segment has the highest share of Vietnamese consumers with university degrees (Table 9).

#### 3.2.7 The anxious

##### 3.2.7.1 Vietnam (26%)

To some extent, at the opposite end of the spectrum, anxious consumers are concerned about all the negative consequences of poor food choices, including animal suffering, environmental depletion, health problems and food safety problems (Tables 5, 7). In contrast with confident consumers, anxious consumers show the lowest share of people holding a university degree amongst the Vietnamese segments (Table 9). Notably, they find it easy to reduce meat intake and establish new eating habits. Furthermore, they are only slightly influenced by the food preferences of other family members (Table 5).

### 3.3 Comparison between both countries

When comparing the mean values over both samples, the Vietnamese appear to be more sensitive to the preferences of their directed surroundings (hindering familial influence) and are subjected to the influence of society as a whole (importance assigned to food that aligns with one’s culture). This sensitivity leads to less modification of eating habits than in Swiss consumers. Price and meat safety are two major concerns for Vietnamese consumers; however, these issues are less of a concern for their Swiss counterparts. This result is arguably directly related to the large differences in wealth levels, food safety standards and types of food outlets between the two countries. Meat consumption behaviour also clearly differs between the two countries. Whilst almost all Vietnamese regularly consume meat, one-third of the Swiss sample describe themselves as flexitarians. It is worth noting that all non-meat consumers were excluded from the study. Our survey results show that at 0.74 kg, the weekly meat consumption is also higher amongst the Vietnamese than amongst the Swiss, who eat an average of 0.51 kg of meat per week. Finally, it should be noted that various socio-demographic variables were found to be significant in either the Vietnamese or Swiss samples. Although gender, place of residence and nationality play a role in Switzerland, household income level is decisive in Vietnam. The share of consumers holding university degrees is the sole variable relevant to both countries.

## 4 Discussion

The evolution of the place and role of meat in the human diet, its symbolic value, long-standing recommendations to eat plenty of meat, relative ease of meat preparation and unique sensory profile contribute to making it a desired food and eating it a pleasure, if not an inalienable right. Recent findings on the health and environmental damage caused by excessive meat consumption and the subsequent progressive adaptations of recommendations struggle to compete with this strong historical and cultural heritage. Changes in behaviour and political decisions are particularly slow and far from sufficient to reach the set of climate goals (Roelfsema et al., 2020; Climate Action Tracker, 2021). Considering the major contributions of meat production and consumption to the climate crisis and overall environmental depletion, this evidence calls for supplementary, strong, multilateral and uncompromising actions.

### 4.1 Discussion of the findings

Switzerland and Vietnam represent two distinct socioeconomic contexts: Western developed countries for the former and emerging economies for the latter. As shown by Whitton et al. (2021), Vietnam’s meat consumption *per capita* is comparable to that of many other emerging countries, whereas Switzerland shares some common characteristics with other developed countries. Thus, our findings can be considered relevant for all regions with socio-economic settings similar to those of the countries studied. This study is amongst the few segmentation research studies on meat consumers that have included both behavioural (e.g., meat consumption quantity and intention to reduce meat consumption) and psychographic factors in their analysis. Psychological factors, such as beliefs, attitude and expectations, play a role in shaping consumers’ preferences and behaviour regarding meat consumption (Font-i-Furnols and Guerrero, 2014). The inclusion of both behavioural and psychographic variables in segmentation studies is essential due to the complexity of food consumption (Verain et al., 2012).

The most interesting parallels can be drawn between our results, particularly those obtained for the Swiss sample, and those of a study conducted in 2019 in the Netherlands by Verain et al. (2022), which also relied on cluster analysis and identified five segments. The Dutch compulsive meat consumers in Verain et al. (2022) share low willingness to reduce meat consumption, meat attachment, and a large percentage of men with both Swiss meat lovers and Swiss basic consumers. The above Dutch compulsive meat consumers are similar to the Vietnamese meat lovers identified in this study, except in terms of gender balance. Australian committed meat eaters identified by Malek et al. (2019) also share certain characteristics with Swiss and Vietnamese meat lovers, such as their high and frequent consumption of meat and the low importance they attach to environmental issues and local foods. These two consumer segments (meat lovers and basic) fit the profile of the archetypal hard-to-influence meat eaters. Our findings imply that, for these meat eaters, high meat attachment is the main barrier to reducing meat consumption. The overrepresentation of men amongst Swiss meat lovers suggests gender differences in meat consumption in Western countries, with men being more attached to meat, as shown by Dowsett et al. (2018).

At the other end of the spectrum, the Swiss proactive in our study resemble the Dutch conscious flexitarians in Verain et al. (2022). Both segments show the lowest meat consumption amongst the respective samples, intend to further reduce said consumption, show sensitivity to environmental issues and animal welfare and are female-dominated. Similar to previous studies (de Boer et al., 2017; Verain and Dagevos, 2022; Valli et al., 2023), our results on the Swiss proactive imply that concerns about the environment and animal welfare are important motives for a low-meat diet. The dominance of women in the Swiss proactive group again suggests gender heterogeneity in meat consumption behaviour. Our results on the Swiss proactive may reflect the fact that women eat less meat and are more open to becoming vegetarians, as shown by Rosenfeld and Tomiyama (2021). However, this gender difference was not observed in the Vietnamese sample.

We found that the Vietnamese proactive share four common features with their Swiss counterparts: moderate meat consumption, high intention to reduce meat consumption, low attachment to meat and a high percentage of flexitarians; nevertheless, their concerns about environmental and animal welfare issues are not as high as those of their Swiss counterparts. This country difference in consumers’ motives regarding meat consumption is in accordance with a study by Markoni et al. (2023), which shows that environmental issues associated with meat are less important to Vietnamese green consumers than to Swiss green consumers. In general, our proactive segment can be compared to Canadian meat reducers (Lacroix and Gifford, 2019) and Australian prospective vegans (Malek et al., 2019) because they all belong to the best-in-class archetypal cluster, which consumes less meat, strives to further reduce meat consumption and has a high awareness of the environmental consequences of meat consumption.

In addition to these two polarised segments, Swiss traditional consumers share certain characteristics with Dutch meat lovers, such as their relative sensitivity to animal welfare and environmental issues despite their average-to-high meat consumption, a moderate attachment to meat and a high proportion of people of national origin. The Swiss and Vietnamese suggestible in our study are similar to each other in many aspects: they have the highest awareness about the health risks of overconsumption of meat, the highest score on hindering familial influence, the second highest intention to reduce meat consumption, a high rating for the importance of animal welfare and environmental issues and a rather low meat intake level. Given these features, they can be placed in an intermediate position in meat consumption, such as the Dutch unconscious flexitarians in Verain et al. (2022). Additionally, the Suggestible segment in our study is fairly close to Dutch unconscious flexitarians in other characteristics, both of which are difficult to characterise and score high on social norms. The suggestible in our study also show some similarities with the Canadian moderate-hinderance meat eaters who held intermediate positions and whose meat reduction efforts seemed to be hindered by a lack of social support (Lacroix and Gifford, 2019). The Swiss suggestible cluster can also be likened to two Australian clusters: willing meat reducers and undecided meat eaters (Malek et al., 2019). In general, our results on suggestible consumers provide evidence of the influence of the social context—particularly the family—on meat consumption. Our results are consistent with those of previous studies (de Boer et al., 2017; Mylan, 2018; Lacroix and Gifford, 2019; Fehér et al., 2020; Collier et al., 2022; Markoni et al., 2023; Valli et al., 2023), which showed that family members can hinder and/or support individual meat reduction. The three archetypal consumer clusters previously identified in the literature (meat lovers, meat reducers and abstainers), although similar to some segments identified in this study to some extent, differ from our segmentation results, which consider cultural factors, such as the difference in perception and consumption of meat in the two distinct cultural settings. However, we argue that a more nuanced view and the inclusion of variables linked to specific barriers (e.g., social norms, cultural influences and social environment) are required to effectively address the issue of meat overconsumption in different geographical settings. For this reason, we do not support the somewhat oversimplified idea of a continuum between committed meat eaters and heavy flexitarians proposed by Verain et al. (2022) but would rather try to understand the specificities of the segments situated between the two extremes as a considerable part of the solution rests on them, that is, on the extent to which they will adapt their behaviour in the near future.

The results for the Swiss traditional and Swiss basics reflect heterogeneity in consumers’ perceptions of meat. The former are concerned about safety, health, the nutritional and cultural dimensions of meat consumption, whereas the latter show little concern about these dimensions. This results in increased meat attachment and consumption levels. These findings suggest that perceptions of the health, nutritional and cultural aspects of meat shape meat consumption in these two segments. Similarly, we found a contradiction between the Vietnamese confident and the Vietnamese anxious. The former are less concerned about meat safety, the health risks of meat consumption, animal welfare and environmental issues and have a low intention to reduce meat consumption. In contrast, the latter express high concern about all these aspects and a high intention to reduce meat consumption. These findings imply that, for these two segments, their perception of the negative consequences of meat consumption might determine their intention to reduce their consumption. The scarcity of literature, particularly segmentation studies on meat consumption in settings comparable to Vietnam (Southern Asian countries or emerging economies), drastically limits our ability to put our results into perspective. However, insights gathered from a preceding qualitative study conducted within the same project (Markoni et al., 2023) and from a study on sustainable consumption in Vietnam (de Koning et al., 2015) confirm our findings, especially regarding the relative importance of the segmentation variables. This, together with the large sample size, leads us to argue that our findings for the Vietnamese sample show some validity for other emerging economies, especially in urban settings.

### 4.2 Practical implications

Eating behaviour and food choices have been shown to have deep roots (Gardner et al., 2011; Rees et al., 2018) and need to be tackled with effective measures. Generic measures such as awareness campaigns help highlight the importance of the subject and keep it on the agenda, but they have been shown to be insufficient on their own to bring about changes in meat consumption behaviour (Bianchi et al., 2018; Osman and Nelson, 2019; Collier et al., 2021)—at least amongst those not already convinced, such as meat lovers and the basic, who are strongly attached to meat and have a low willingness to reduce their meat consumption. Previous studies (Hielkema and Lund, 2021; Verain et al., 2022) highlighted that these groups deliberately ignore the information given. Because these two segments do not place much importance on animal welfare or environmental issues, one potential strategy would be to focus on a topic that concerns them more: health benefits. Thus, for meat lovers and the basic, the campaign could focus on the real and scientifically proven effects of a low-meat diet on health (e.g., through testimonials from citizens who have reduced the risk of developing a disease after changing their diet) and present examples of plant-based foods that strengthen the body and provide energy in an appropriate way [e.g., using vegetarian or vegan sports people or people of influence as ambassadors (Stoll-Kleemann and Schmidt, 2017; Hielkema and Lund, 2021)]. Moreover, the campaign should visualise the recommended weekly consumption of food groups (see, for example, Vermeulen, 2019) and refer to resources (e.g., websites, cooking videos, flyers at retailers) that provide concrete advice on how to cook various quick and healthy dishes with less meat (Mullee et al., 2017; Kemper, 2020; Vermeulen et al., 2020; Collier et al., 2021) without compromising taste and convenience, whilst considering local eating habits and traditions.

Addressing the lack of skills and experience in practising a low-meat diet, the campaign can empower meat lovers and the confident, whose perceived self-efficacy in meat reduction is currently very low. Such campaigns should be presented in a positive light, spreading the idea that change is easy to achieve and beneficial for everyone, including future generations. It should also aim to deconstruct certain misconceptions about the benefits of meat (as a source of strength and vigour), the appropriateness of current portion sizes and meat consumption frequency and the insufficient satiating effect of meat-free menus. An emotional message entitled “less but better,” which encourages conscious meat consumption (that is, smaller quantities of healthier, more sustainable meat), could be a way of approaching the proactive, the anxious and above all the traditional, who are difficult to reach. All three segments are sensitive to sustainability arguments; however, to tangibly influence the behaviour of the less convinced and less impressionable segments (the traditional, the basic, the confident and the meat lovers), governments should be pro-active and more responsible and use all levers within their sphere of influence, such as the use of regulatory measures (Verplanken and Whitmarsh, 2021) or the involvement or even compulsion of other stakeholders to participate in the change towards a more plant-based diet. In practice, this could start by offering a standard vegetarian menu (default option), introducing meat-free days, focussing on less impactful meat types and cuts, adapting portion sizes and introducing price differentiation according to the absence or presence of meat (or fish) on the menu in all state-run and subsidised canteens. These measures would not only directly contribute to reducing meat consumption, but they can also set new social norms and inspire customers on how to cook with less meat.

Education is another powerful instrument as it offers a unique opportunity to reach an entire population at an early age when habits can still be influenced. In Switzerland, almost all teenagers take home economics classes as a part of their compulsory education. It is a great opportunity to talk about meat consumption and its consequences; compare the environmental and health costs of different types of diets; understand why it is important to consume all parts of an animal; learn how to minimise food waste; (re)learn what a suitable portion of meat is; gain experience and confidence in preparing meatless meals, meat substitutes, second cuts and offal; and challenge and reinvent traditions and festive menus. Teenagers could act as gateways for their households, stimulating discussion and possibly influencing family practices. Unfortunately, in Switzerland, compulsory schooling is not a national but a cantonal responsibility, meaning that the decision to include specific subjects in programmes cannot be taken at the national level. Environmental considerations should also be taken into account when formulating official national dietary recommendations, such as the food pyramid and ideal plate, following the model developed by the EAT-Lancet Commission on healthy diets from sustainable food systems (Willett et al., 2019) as did Fesenfeld et al. (2023) for Switzerland.

Regarding regulatory measures, the revision of agricultural subsidy systems is a priority in several developed countries. This would rebalance the final prices of animal-and plant-based foods, making them financially attractive. Additional taxes on meat that reflect the environmental costs of its production and consider regional differences (i.e., climate and topography) might be necessary to reach a larger share of the population. The introduction of a unique official mandatory environmental label for all food products is another lever to consider given its proven beneficial effects (Storcksdieck genannt Bonsmann et al., 2020; Roberto et al., 2021). Another potentially powerful lever lies in a government’s ability to involve other influential players in the collective effort towards reduction in meat consumption. In concrete terms, this means inviting representatives of the retail and private catering sectors to make a formal commitment to systematically promote plant-based foods (in terms of positioning at points of sale and on menus, price reductions, special offers, tastings and so on) and to renounce all forms of meat promotion. Finally, the government can stimulate innovation and research in a targeted manner by creating special calls for tenders with dedicated budgets. Because meat attachment is high across most consumer segments, the development of tasty, convenient and minimally processed meat substitutes can considerably assist meat reduction; therefore, the development of novel meat alternatives may be useful for future research.

### 4.3 Limitations

The method used to recruit participants for the Vietnamese sample (snowballing principle starting from employees of universities and large companies working in urban areas) aims to focus on a population for whom meat reduction could be a topical issue, who do not suffer from deprivation due to insufficient financial means and who have a certain amount of choice when it comes to food and supply sources. This approach has consequences in terms of representativeness, which appears to be evident when comparing the education and income levels of the sample with national statistics. The segmentation of the Vietnamese sample can, therefore, be considered representative of the urban Vietnamese population and possibly of other urban populations from South-East Asia and other emerging economies. Similarly, the proposed measures and actions are primarily aimed at affluent urban dwellers from (southeast Asian) emerging economies and inhabitants of Westernised developed countries. This is also in view of the fact that, to date, no study has demonstrated a link between meat consumption and adverse health effects in Asian populations.

Wherever possible, validated scales were used to measure the constructs used in the segmentation. However, the choice of scales was limited by the nature of the sample, which includes meat consumers from two different cultural settings. Some validated scales were considered unsuitable or incomprehensible for part of the target population and had to be replaced by new ones.

## 5 Conclusion

This study sheds light on the intentions, drivers and barriers to meat consumption that characterise the different consumer segments making up the population of a developed Western country and the urban population of an emerging economy. Surprisingly, the results of the cluster analyses conducted on both the Swiss and Vietnamese samples showed numerous parallels. Three of the five clusters disclosed in both samples, representing approximately 60% of the respective populations, are found in both countries and thus probably in many similar settings. This outcome allowed us to develop a set of recommendations for action that may be valid in comparable socio-economic and cultural contexts.

Based on the psychographic profiles of the studied populations and previous research, we argue that communication and raising awareness are required, especially in the context of emerging economies, but are not sufficient on their own to induce behavioural changes in meat consumption. To achieve the target of a planetary health diet, relying on nudging techniques and the voluntary actions of private actors will not be sufficient. Governments must, therefore, shoulder their responsibilities and pull all the levers at their disposal, including overhauling the food on offer in all state-run or subsidised restaurants, using a portfolio of regulatory measures and requiring private sector actors to participate in the effort and modify their offer accordingly. Several of these measures correspond to profound paradigm changes and will encounter political headwinds; resistance from the industry, retail and catering sectors; and even possibly from the populations of countries, as Richter et al. (2022) highlighted in their study on the acceptance of meat reduction policies in Switzerland. Thus, the best approach is likely to commit to a common international agenda, agreeing on short-term goals to achieve and a common set of measures to implement. Finally, further segmentation studies from emerging economies would be beneficial and help lend weight to our conclusions.

## Data availability statement

The data presented in the study and statistical analyses generated for this study are deposited in the OLOS repository: https://doi.org/10.34914/olos:72wgbsi5ajdljmomf3msxikq5e.

## Ethics statement

Ethical approval was not required for this study. Data were collected anonymously through online surveys; the topic is not sensitive. The studies were conducted in accordance with the local legislations and institutional requirements. The participants provided their written informed consent to participate in this study.

## Author contributions

MD: Conceptualisation, Data curation, Formal analysis, Investigation, Methodology, Project administration, Supervision, Validation, Visualisation, Writing – original draft, Writing – review & editing. TMH: Conceptualisation, Data curation, Funding acquisition, Investigation, Methodology, Project administration, Supervision, Validation, Writing – review & editing. FG: Conceptualisation, Funding acquisition, Methodology, Validation, Writing – review & editing, Project administration. EM: Funding acquisition, Methodology, Writing – review & editing, Conceptualisation, Project administration. MHN: Conceptualisation, Funding acquisition, Investigation, Methodology, Validation, Writing – review & editing. ADN: Conceptualisation, Funding acquisition, Methodology, Writing – review & editing, Investigation. TLB: Funding acquisition, Writing – review & editing, Project administration, Investigation. NTL: Funding acquisition, Writing – review & editing, Investigation. BDP: Funding acquisition, Project administration, Supervision, Writing – review & editing, Investigation. TAB: Conceptualisation, Funding acquisition, Methodology, Project administration, Supervision, Writing – review & editing.
